# Real‐time detection of volatile metabolites enabling species‐level discrimination of bacterial biofilms associated with wound infection

**DOI:** 10.1111/jam.15313

**Published:** 2021-10-19

**Authors:** Elisabeth A. Slade, Robin M. S. Thorn, Amber E. Young, Darren M. Reynolds

**Affiliations:** ^1^ Centre for Research in Biosciences University of the West of England Bristol UK; ^2^ Bristol Centre for Surgical Research Population Health Sciences Bristol Medical School University of Bristol Bristol UK

**Keywords:** biofilms, diagnosis, pseudomonads, staphylococci, streptococci

## Abstract

**Aims:**

The main aim of this study was to investigate the real‐time detection of volatile metabolites for the species‐level discrimination of pathogens associated with clinically relevant wound infection, when grown in a collagen wound biofilm model.

**Methods and Results:**

This work shows that *Staphylococcus aureus*, *Pseudomonas aeruginosa* and *Streptococcus pyogenes* produce a multitude of volatile compounds when grown as biofilms in a collagen‐based biofilm model. The real‐time detection of these complex volatile profiles using selected ion flow tube mass spectrometry and the use of multivariate statistical analysis on the resulting data can be used to successfully differentiate between the pathogens studied.

**Conclusions:**

The range of bacterial volatile compounds detected between the species studied vary and are distinct. Discrimination between bacterial species using real‐time detection of volatile metabolites and multivariate statistical analysis was successfully demonstrated.

**Significance and Impact of the Study:**

Development of rapid point‐of‐care diagnostics for wound infection would improve diagnosis and patient care. Such technological approaches would also facilitate the appropriate use of antimicrobials, minimizing the emergence of antimicrobial resistance. This study further develops the use of volatile metabolite detection as a new diagnostic approach for wound infection.

## INTRODUCTION

Biofilms are complex communities of micro‐organisms attached to biotic or abiotic surfaces, and are encased in a primarily self‐produced extracellular matrix (Hall & Mah, 2017; Roy et al., 2018), comprised of polysaccharides, proteins, lipids and extracellular DNA (Vyas & Wong, 2016). Biofilm tolerance to antimicrobials has been widely reported, whereby contributing mechanisms include antimicrobial agent interaction with the extracellular matrix inhibiting penetration; slow growth rate resulting in reduced susceptibility; heterogeneous metabolism and the presence of persisters (Olsen, 2015). The concentration of antibiotics required to treat biofilm infections has been shown to be 100‐ to 1000‐fold higher than the planktonic minimum inhibitory concentration; consequently, biofilm infections are likely to persist long term despite antibiotic treatment, potentially resulting in relapse after treatment is completed (Del Pozo, 2018; Hall & Mah, 2017).

The presence of bacterial biofilms within the wound bed has been observed in 78.2% of chronic wounds, and are thought to play a crucial role in delayed wound healing, the key characteristic of the chronic wound state (Malone et al., 2017). Biofilm infection stimulates a prolonged inflammatory response, resulting in an increase in wound exudate production, which, in turn, provides a source of nutrients to support the increasing bacterial population (Hughes, 2016).

Diagnosis of wound infection relies on the ability of clinicians to identify the clinical signs of infection, supported by the use of non‐specific blood tests for markers of inflammation (Blokhuis‐Arkes et al., 2015). The signs of infection include increased pain, an excessive or increased volume of exudate, localized heat and swelling, malodour and erythema (Hughes, 2016; Macgregor et al., 2008). There is no definitive test available to identify wound infection, and microbiological wound cultures are employed to supplement clinical diagnosis and inform treatment at a later date (Blokhuis‐Arkes et al., 2015; Greenhalgh et al., 2007). However, the bacterial load or type detected cannot provide a diagnosis in itself and microbiological analysis should only be used to confirm the presence of pathogenic strains within the wound bed and establish antibiotic sensitivities (Edwards & Harding, 2004; International Wound Infection Institute, 2016; Landis, 2008; Macgregor et al., 2008; Sibbald et al., 2003). Diagnosis of wound infection is therefore still subjective, relying on the experience of the clinician and their interpretation of clinical symptoms, signs and laboratory test results. If the microbial burden of wounds is not adequately managed, then there is a risk of the development of sepsis. This is a serious complication and is the leading cause of mortality in patients with severe burn wounds (Ma et al., 2016; Patil et al., 2017; White et al., 2015). Sepsis‐related deaths account for 50%–84% of deaths in adult patients with severe burns and 55% of deaths in paediatric burns patients (Lopez et al., 2017).

Early management of clinically relevant wound infection is essential to minimize the risk of sepsis (Ma et al., 2016; Patil et al., 2017; White et al., 2015). However, isolation and susceptibility testing of infecting organisms can take several days, often resulting in the empirical prescribing of antibiotics (Retamar et al., 2012). The UK government commissioned ‘O’Neill review’ on antimicrobial resistance (O’Neill, 2016) highlighted the common practice of empirical prescribing of antimicrobials as a major concern, leading to huge over‐use of antibiotics, and consequently contributing to rising antimicrobial resistance. The report recommends promoting development of new, rapid diagnostic techniques as a key strategy to overcome the unnecessary use of antimicrobials. Point‐of‐care diagnostics have the potential to revolutionize the management and treatment of wound infections. For example, the ability to rule out infection would prevent over‐use of antimicrobials when there is no clinical need, thereby helping to reduce the emergence of antimicrobial resistance. In addition, early identification of the causative organism where an infection is identified, combined with knowledge of local epidemiology, would expedite selection of the most appropriate drug, improving patient outcomes.

A novel approach to rapid wound diagnostics could be the use of microbial volatiles as a means of identification. Bacteria produce volatile compounds, that is, metabolites with a low molecular weight and high vapour pressure that are released into the surrounding environment. Over 1000 bacterially derived volatile compounds have been described, with a single species capable of producing up to 80 different compounds (Audrain et al., 2015; Schulz & Dickschat, 2007). It is well known that metabolism of growth substrates (e.g. C, N, S) differs between bacterial species, due to the inherent variability in genomic expression of different metabolic pathways. However, this also results in the production of different volatile compounds, the profiling of which has been shown to be species‐specific in vitro (Chippendale et al., 2014; Thorn & Greenman, 2012; Thorn et al., 2011).

The use of bacterial volatile metabolites as a diagnostic tool has been previously demonstrated for lung infections (Dryahina et al., 2016; Fowler et al., 2015; Kramer et al., 2015; Nizio et al., 2016; Purcaro et al., 2018), whereby respiratory pathogens in vitro (Dryahina et al., 2016; Nizio et al., 2016; Purcaro et al., 2018) and in vivo (Fowler et al., 2015; Lewis et al., 2017) were discriminated based upon the profile of volatile metabolites detected. Biofilm volatiles have also been previously investigated, but primarily in the field of oral microbiology, whereby malodour studies have focussed on volatile sulphur‐compound‐producing species (Greenman et al., 2013; Krespi et al., 2006; Washio et al., 2005) and the effects of oral malodour treatments (Greenman et al., 2013; Saad et al., 2012, 2013). Few studies exist in the current literature that are concerned with the detection and analysis of wound volatiles. Thomas et al. (2010) used skin patches to collect volatile samples from chronic skin lesions and healthy skin, prior to GC‐MS analysis. The authors found statistically significant differences between the volatile profiles of healthy skin compared to those of chronic wounds, but these differences were not directly attributed to wound microbiology (Thomas et al., 2010). Recent research has demonstrated the detection of volatile profiles for discrimination between pathogens associated with wound infection, in planktonic culture in vitro, using both complex culture media and a simulated wound fluid (Slade et al., 2017). Other recent research has demonstrated the detection of species‐specific volatile profiles using GC‐MS analysis of an ex vivo biofilm model (Ashrafi et al., 2018). However, this study used a static model system, utilizing complex growth media, and applied only limited statistical analysis on the resultant volatile data produced.

Gas chromatography mass spectrometry (GC‐MS) is used extensively for the analysis of volatile compounds and combines chromatographic separation of analyte compounds with mass spectral analysis for identification (Langford et al., 2014). Selected ion flow tube‐mass spectrometry (SIFT‐MS) facilitates real‐time detection and quantification of volatile compounds in humid air samples, whereby reagent ions (H_3_O^+^, NO^+^ and O_2_^+^) generated in a gas ion discharge source and selected by a quadrupole mass filter are injected into a fast flowing helium carrier gas in the reaction flow tube. The sample gas is introduced into a flow tube via a heated sample inlet, where chemical ionization occurs resulting in the production of characteristic product ions. Downstream, reagent and product ions are separated and counted by a further quadrupole mass spectrometer and electron multiplier detector system. Finally, absolute concentrations of trace gases can be quantified based on the ratios of ion count rates and the previously determined reaction rate constants contained within the integrated kinetics library (Smith & Španěl, 2011, 2015; Španěl et al., 2006).

GC‐MS is considered the best analytical technique for the identification of volatile compounds, particularly in complex mixtures. However, GC‐MS analysis can be time‐consuming and requires pre‐concentration of samples such as on solid phase micro extraction (SPME) fibres or thermal desorption tubes (Shestivska et al., 2012; Zscheppank et al., 2014). In contrast, SIFT‐MS analysis is a direct mass spectrometry technique, so does not require any sample preparation. However, SIFT‐MS does have limitations for volatile analysis, for instance, in a mixture containing a range of analytes it is likely that a number of product ions may have identical masses, and so the reaction with that particular reagent ion must be omitted from any calculation to derive analyte concentration (Langford et al., 2014). A combined approach, using GC‐MS for reliable compound identification and SIFT‐MS for real‐time quantification, exploits the advantages of both techniques.

The main aim of this study was to determine whether three commonly isolated bacterial pathogens associated with clinically relevant wound infection (*Staphylococcus aureus*, *Pseudomonas aeruginosa* and *Streptococcus pyogenes*) could be successfully discriminated based on their production of volatile metabolites when grown in a collagen wound biofilm model. The model described enables continuous supply of substrates for metabolism and removal of waste products, resulting in steady‐state microbial biofilms which are more representative of the in vivo environment (Slade et al., 2019). Volatile analysis was undertaken using SIFT‐MS coupled with multivariate data analysis for the detection and discrimination of bacterial biofilms. The work presented here lays the foundations for the potential development of rapid point‐of‐care microbial diagnostics based on volatile detection, where biofilms are known to be a significant cause of morbidity and mortality.

## MATERIALS AND METHODS

### Preparation and maintenance of bacterial cultures

Bacterial cultures were maintained on beads (Microbank; Pro Lab Diagnostics) at −80°C, resuscitated as required on Tryptone Soya Agar (Oxoid) or blood agar (Oxoid) and incubated aerobically at 37°C. Working cultures were stored on sealed plates at 4°C. The following bacterial strains were used during this study: *P*. *aeruginosa* NCIMB 10548, NCIMB 8295 and ATCC 15442; *Staph. aureus* NCIMB 6571, ATCC 6538 and a clinical strain of methicillin‐resistant *Staph*. *aureus* (obtained from Southmead Hospital); *Strep. pyogenes* NCTC 10881, NCTC 10874 and NCTC 10871.

### Growth of bacterial biofilms in a collagen wound biofilm model

Biofilms were cultured for 48 h in a collagen wound biofilm model developed previously (Slade et al., 2019). A high concentration Type I collagen gel from rat tail (Corning Incorporated) was neutralized to pH 7 and diluted to 2.0 mg ml^−1^ with simulated wound fluid (1:1; FBS: 0.1% peptone and 0.85% NaCl). Sterile microscope slides were coated with 1.5 ml of the resulting solution and allowed to polymerize at 37°C for 1 h.

Overnight plate cultures (18–24 h) were used to prepare suspensions of the test organisms in 10 ml simulated wound fluid adjusted to an OD_620nm_ of 0.20. One millilitre of each bacterial suspension was used to inoculate a collagen‐coated microscope slide housed within a sterile Petri dish. The inoculated slides were incubated at 33°C for 2 h to allow adherence of organisms.

Inoculated collagen‐coated slides were aseptically transferred to the channels of the drip flow biofilm reactor, whereupon tubing from the media reservoir and to waste collection were connected and the reactor incubated at 33°C for 48 h with a SWF flow rate of 2 ml h^−1^.

### SIFT‐MS analysis of bacterial biofilms

Volatile compounds were sampled from the headspace of drip flow biofilm reactor channels by connecting the heated direct sample inlet of the SIFT‐MS instrument (Voice200Ultra; Syft Technologies) to the reactor channel via a 45 cm length of PEEK tubing (Supelco) of OD 1/16 in. × I.D. 0.030 in. First, the SIFT‐MS instrument was operated in Full Scan Mode (FS) using the H_3_O^+^ reagent ion and volatile compound product ion peaks detected over a spectrum range of 10–200 *m*/*z*. Three replicate scans were obtained from each biofilm sample and three independent biofilms of each strain analysed. Second, following GC‐MS to identify compounds of interest, the SIFT‐MS instrument was operated in selected ion mode (SIM). The H_3_O^+^, NO^+^ and O2^+^ reagent ions were used to quantify the concentration of selected compounds in the bacterial biofilm headspace. Each biofilm sample was scanned for 60 s, resulting in a total of 12 replicate measurements per compound, and three independent biofilms of each bacterial strain were analysed.

### HS‐SPME‐GCMS analysis of bacterial biofilms

Volatile compounds were sampled from the headspace of drip flow biofilm reactor channels by SPME using a 75 µm Carboxen/Polydimethylsiloxane (CAR/PDMS) fibre assembly for 30 min at 33°C. After sampling, the fibre was retracted and transported in a sealed container to the GC‐MS instrument and immediately inserted into the heated inlet. The GC (6890N; Agilent Technologies) was programmed with the following method: splitless injection, inlet temperature held at 305°C, helium carrier gas flow rate 1 ml min^−1^, oven temperature programme set to 35°C for 3 min, then 4°C per minute ramp up to 100°C followed by 8°C per minute ramp up to 300°C with a 5‐min hold at the final temperature. A 25 m, 5% phenyl‐methylpolysiloxane column (HP‐5ms; Agilent Technologies) was used. The mass selective detector (5973 MSD; Agilent Technologies) was used in ‘Full Scan Mode’ to detect ions over a spectrum range of 15–400 *m*/*z*. Analysis software (Agilent MassHunter Workstation software) was used for compound identification using chromatogram deconvolution algorithms and comparison with the NIST2.0 library.

### Data analysis

The FS datasets were generated by performing three replicate scans of each biofilm sample and the SIM scans generated 12 replicate measurements per compound for each biofilm sample. The mean product ion intensities or compound concentrations of these replicate scans were calculated for each independent biofilm. Subsequently, the FS data were square root transformed and the SIM data subject to log base 10 (log10) transformation prior to the analysis described below. Log10 transformation is used to normalize skewed datasets. However, log10 transformation is not suitable for datasets that contain zero values, as was the case for the FS dataset here, so square root transformation was used as an alternative.

To identify the SIFT‐MS product ion peaks from the full scan data that are associated with species discrimination, a multivariate analysis of variance (MANOVA) was performed to identify product ion peaks with significantly different (*p* < 0.01) peak intensities between species. Similarly, MANOVA was used to identify which compounds from the SIM scan data were measured at significantly different (*p* < 0.01) concentrations between species. The MANOVA was used to identify if differences in each product ion peak intensity or compound concentration were present between species, with the aim of identifying product ion peaks or compounds suitable for targeting species discrimination. However, this approach did not identify between which species these differences were observed. Further analysis using hierarchical cluster analysis and principal component analysis (PCA) was then undertaken, using only the data for product ion peaks (FS) or compounds (SIM) identified as significantly different between species by MANOVA.

Hierarchical cluster analysis was used to identify groupings within each dataset, visualized by the production of a dendrogram, which indicates the relationship between the clusters of samples. PCA was also used to facilitate simple visualization, transforming the variables to principal components, which retain the majority of the variability from the original dataset within the first few principal components. Plotting the scores for the samples against the first two principal components provides a two‐dimensional summary of the data, which highlights grouping of samples. In addition, scatter plots of the loading of the original variables, on the first two principal components, indicate the influence of these variables within the model. Comparison of the two plots indicates the *m*/*z* peaks (FS) or compounds (SIM) that are most important for separating the groups of samples (i.e. bacterial species).

Initial data processing was carried out using Microsoft Excel 2016 (Microsoft Corporation) and MANOVA, hierarchical cluster analysis and PCA utilized IBM SPSS Statistics versions 25 (IBM Corporation).

See supporting information (Figure S1) for a flow diagram of the methodology and statistical analysis.

## RESULTS

### Full scan SIFT‐MS analysis of biofilm samples

**FIGURE 1:**
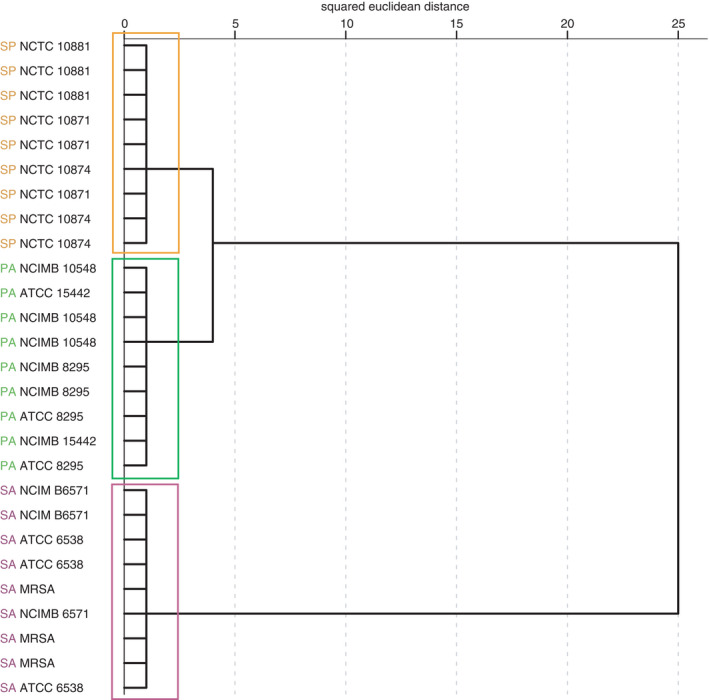
Dendrogram generated by hierarchical cluster analysis using 59 selected headspace volatile product ion peaks (√cps), detected by SIFT‐MS following 48 h of continuous culture in the collagen wound biofilm model (*n* = 3 biofilms per strain, with three replicate scans per biofilm). Coloured boxes indicate species‐specific clustering. *Pseudomonas aeruginosa* (green), *Staphylococcus aureus* (pink), *Streptococcus pyogenes* (orange) [Colour figure can be viewed at wileyonlinelibrary.com]

Figure 1 shows the dendrogram generated to visualize hierarchical cluster analysis of the FS SIFT‐MS analysis of bacterial biofilms. The dendrogram shows clear discrimination between *Staph*. *aureus*, *Strep*. *pyogenes* and *P*. *aeruginosa* biofilms at a species level, based on the profile of the 59 product ion peaks selected using MANOVA.

**FIGURE 2:**
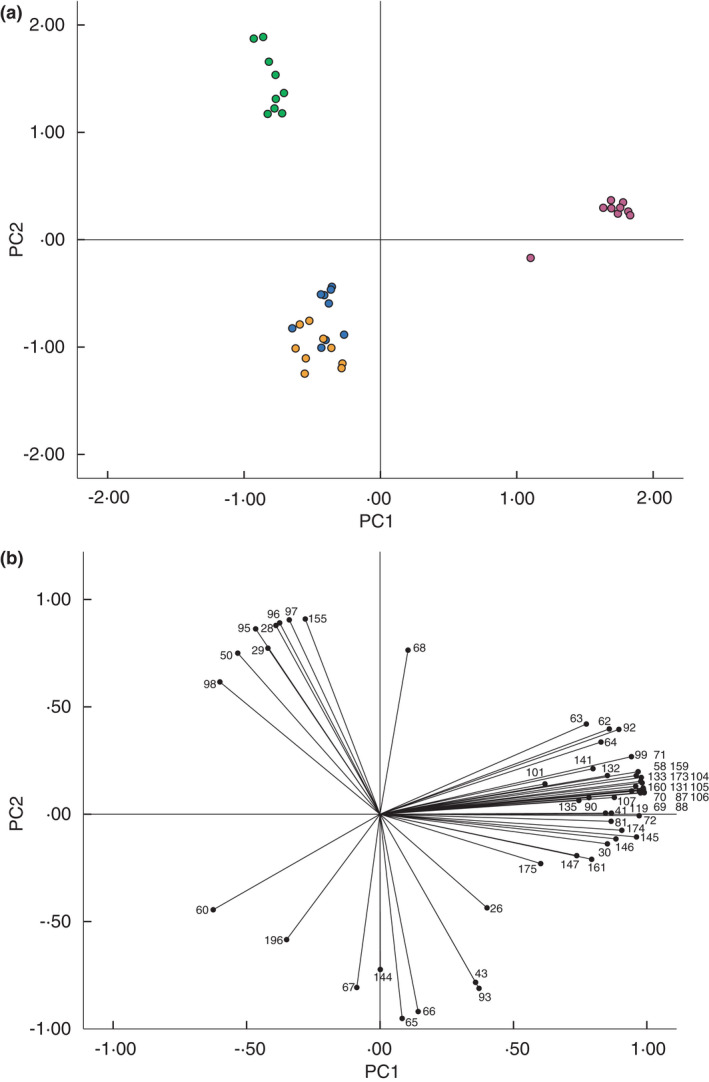
(a) Plot of the scores of the first two principal components generated by principle component analysis of SIFT‐MS full scan data of selected headspace volatiles product ion peaks of bacterial biofilms and uninoculated controls (*n* = 3 scans per sample). Principal component 1 (horizontal axis) accounts for 57.7% of the total variation in the original dataset and principal component 2 (vertical axis) accounts for 22.6% of the total variation. *Pseudomonas aeruginosa* (green), *Staphylococcus aureus* (pink), *Streptococcus pyogenes* (orange), control (blue). (b) Principal component analysis (PCA) loading plot of selected m/z peaks. Data points indicate the loading of each *m*/*z* peak (variable) on the first two principal components generated from the PCA [Colour figure can be viewed at wileyonlinelibrary.com]

Figure 2a shows a scatter plot of the scores for each independent biofilm against the first two principal components generated from PCA of the 59 product ion peaks selected using MANOVA. The first two principal components account for a total of 80.3% of the variability within the original dataset. The scatter plot (Figure 2a) shows clear discrimination between *Staph*. *aureus*, *Strep*. *pyogenes* and *P*. *aeruginosa* biofilms. However, inclusion of the controls (uninoculated) shows substantial overlap between *Strep*. *pyogenes* biofilms and the control samples. Figure 2b shows the loading of each of the original variables (SIFT‐MS product ion peaks) on the first two principal components and indicates the influence of each of these variables on the positioning of the samples in Figure 2a. Only three product ions are located within the region of the plot associated with the position of *Strep*. *pyogenes* and the uninoculated controls (60, 67 and 196 *m*/*z*). Eight product ions (28, 29, 50, 95, 96, 97, 98 and 115 *m*/*z*) influence the position of *P*. *aeruginosa* on the PCA plot, while up to 41 product ions are involved in determining the position of the *Staph*. *aureus* biofilms.

**FIGURE 3:**
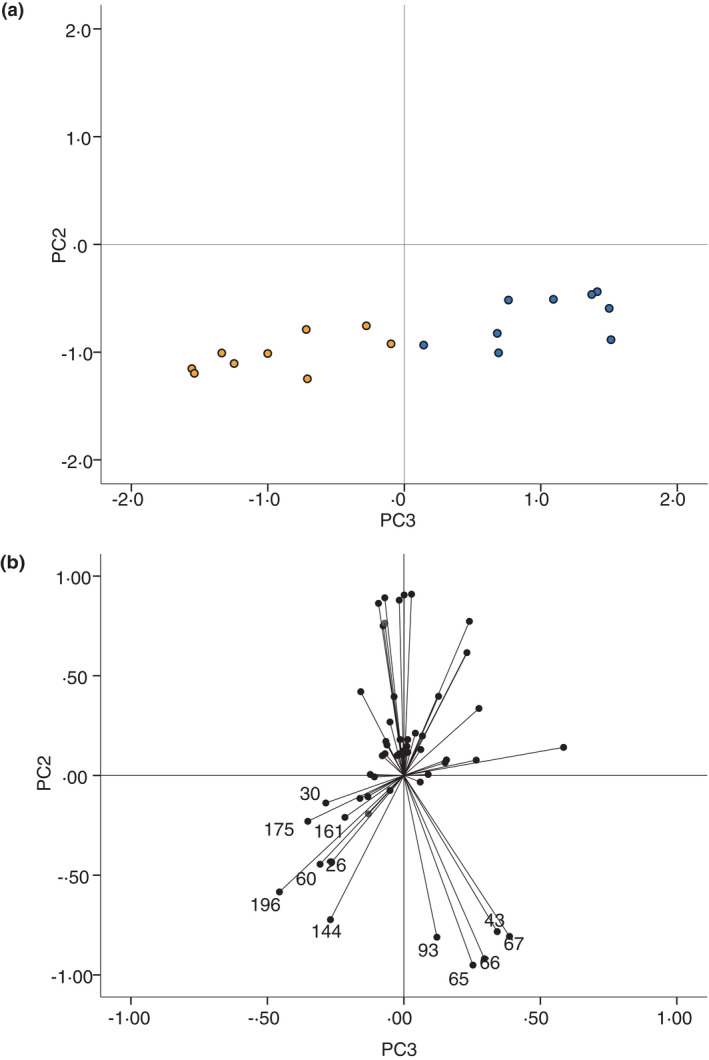
(a) Plot of the scores of the second and third principal components generated by principle component analysis of SIFT‐MS full scan data. *Streptococcus pyogenes* (orange) and control (blue) only are shown. The second principal component accounts for 22.6% and the third components accounts for 3.67% of the total variation of the original dataset. (b) Principal component analysis (PCA) loading plot of selected *m*/*z* peaks. Data points indicate the loading of each *m*/*z* peak (variable) on the second and third principal components generated by PCA [Colour figure can be viewed at wileyonlinelibrary.com]

Although the first two principal components account for the majority (80.3%) of the total variation within the dataset of 59 selected product ions, the inclusion of the third principal component increases this further (by 3.67%) and allows for a 3D plot of the first three components to be constructed. Figure 3 shows a plot of only the second and third principal components, showing that the *Strep*. *pyogenes* and control samples can clearly be differentiated. Figure 3b suggests that the product ions most significantly influencing the positioning of *Strep*. *pyogenes* on the PCA plot are 26, 30, 60, 144, 161, 175 and 196 *m*/*z* and that 43, 65, 66, 67 and 93 *m*/*z* have the strongest influence on the positioning of the control samples.

### HS‐SPME‐GCMS analysis of biofilm samples

Table 1 lists the compounds identified in the headspace of bacterial biofilm samples by GC‐MS using the chromatogram deconvolution function within the Agilent mass hunter software followed by comparison with the NIST 2.0 library. Alongside the general compound information, the SIFT‐MS product ions used for subsequent quantification of each of the compounds by SIFT‐MS are listed. Following identification of compounds of interest using HS‐SPME‐GCMS, SIFT‐MS analysis using SIM was performed on three independent biofilm samples of each strain of bacteria used in the study, to enable quantification of the compounds listed in Table 1. In addition to the 17 compounds identified using GC‐MS, hydrogen cyanide and ammonia were also included in the SIFT‐MS SIM scan analysis, due to consistent reporting in the literature of the importance of these compounds as potential biomarkers of *P*. *aeruginosa* (Gilchrist et al., 2013; Neerincx et al., 2016; Smith et al., 2013). In addition, we have previously detected both hydrogen cyanide and ammonia from *P*. *aeruginosa* biofilms cultured in the collagen wound biofilm model throughout biofilm growth and development (Slade et al., 2019). Hence, 19 compounds in total were included for quantification using SIFT‐MS SIM scans.

**TABLE 1:** Table of compounds detected by HS‐SPME‐GCMS from bacterial biofilms cultured in the collagen wound biofilm model for 48 h. Corresponding product ions for quantification (SIFT‐MS) and associated bacterial species are shown; SP, *Streptococcus pyogenes*; PA, *Pseudomonas aeruginosa*; SA, *Staphylococcus aureus*. References indicate other studies where these compounds have been detected in the headspace of bacterial cultures

Compound	Molecular formula	Molecular weight	Functional group	Species			SIFT‐MS product ions			References
				SP	PA	SA	H_3_O^+^	NO^+^	O_2_^+^	
Ethanol	C_2_H_6_O	46.08	Alcohol	✓	✓	✓	47	45		Thorn et al., 2011; Shestivska et al., 2012; Filipiak et al., 2012
1‐butanol	C_4_H_10_O	74.14	Alcohol	✓			57	73	56	Thorn et al., 2011
Isobutyl alcohol	C_4_H_10_O	74.14	Alcohol	✓		✓	57	73	42	Shestivska et al., 2012
2‐methyl−2‐propanol	C_4_H_10_O	74.14	Alcohol	✓			57	57	59	Filipiak et al., 2012
3‐methyl−1‐butanol	C_5_H_12_O	88.17	Alcohol			✓	71	87	59	Filipiak et al., 2012
Acetaldehyde	C_2_H_4_O	44.06	Aldehyde	✓			45	43		Thorn et al., 2011; Shestivska et al., 2012; Filipiak et al., 2012
2‐methylpropanal	C_4_H_8_O	72.12	Aldehyde			✓		71	72	Thorn et al., 2011; Filipiak et al., 2012
2‐methylbutanal	C_5_H_10_O	86.15	Aldehyde			✓	87	85	58	Thorn et al., 2011
3‐methylbutanal	C_5_H_10_O	86.15	Aldehyde			✓	87	85		Filipiak et al., 2012
Acetoin	C_4_H_8_O_2_	88.11	Ketone			✓	89	118	88	Thorn et al., 2011; Filipiak et al., 2012
Acetone	C_3_H_6_O	58.09	Ketone	✓	✓	✓	59	88		Thorn et al., 2011; Shestivska et al., 2012
Butanone	C_4_H_8_O	72.12	Ketone	✓		✓		102	72	Shestivska et al., 2012; Filipiak et al., 2012
2,3‐butanedione	C_4_H_6_O_2_	86.09	Ketone	✓		✓	87	86	86	Filipiak et al., 2012
3‐methylbutanoic acid	C_5_H_10_O_2_	102.15	Carboxylic acid	✓			103	132		Filipiak et al., 2012
Butane	C_4_H_10_	58.15	Alkane		✓				43	Filipiak et al., 2012
Dimethyl sulphide	C_2_H_6_S	62.15	Sulphur compound		✓		63	62	62	Shestivska et al., 2012; Filipiak et al., 2012
Dimethyl disulphide	C_2_H_6_S_2_	94.21	Sulphur compound		✓		95	94	94	Thorn et al., 2011; Shestivska et al., 2012; Filipiak et al., 2012

### Selected ion mode SIFT‐MS analysis of biofilm samples

**FIGURE 4:**
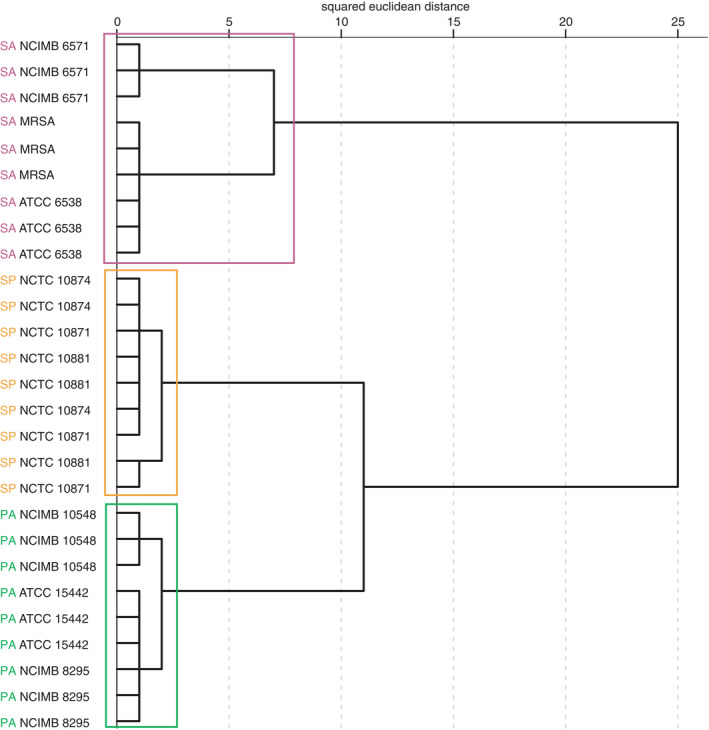
Dendrogram generated by hierarchical cluster using eight selected headspace volatile compound concentrations (ppb), detected by SIFT‐MS following 48 h of continuous culture in the collagen wound biofilm model. (*n* = 3 biofilms per strain, with 12 replicate scans per biofilm). Coloured boxes indicate species‐specific clustering. *Pseudomonas aeruginosa* (green), *Staphylococcus aureus* (pink), *Streptococcus pyogenes* (orange) [Colour figure can be viewed at wileyonlinelibrary.com]

Of the 19 compounds quantified from bacterial biofilms using SIFT‐MS SIM analysis, eight were found to differ significantly (*p* < 0.01) between bacterial species using MANOVA. These were hydrogen cyanide, dimethyl sulphide, acetaldehyde, 3‐methyl‐1‐butanol, 2,3‐buanedione, 2‐methyl‐2‐propanol, 2‐methylbutanal and 3‐methylbutanal. Hierarchical cluster analysis was applied to the data from these eight volatiles alone, resulting in the dendrogram shown in Figure 4. The dendrogram shows clear discrimination between *Staph*. *aureus*, *Strep*. *pyogenes and P*. *aeruginosa* biofilms based on SIFT‐MS quantification, using these eight identified volatile compounds, detected in the sample headspace after 48 h of continuous culture in the collagen wound biofilm model.

**FIGURE 5:**
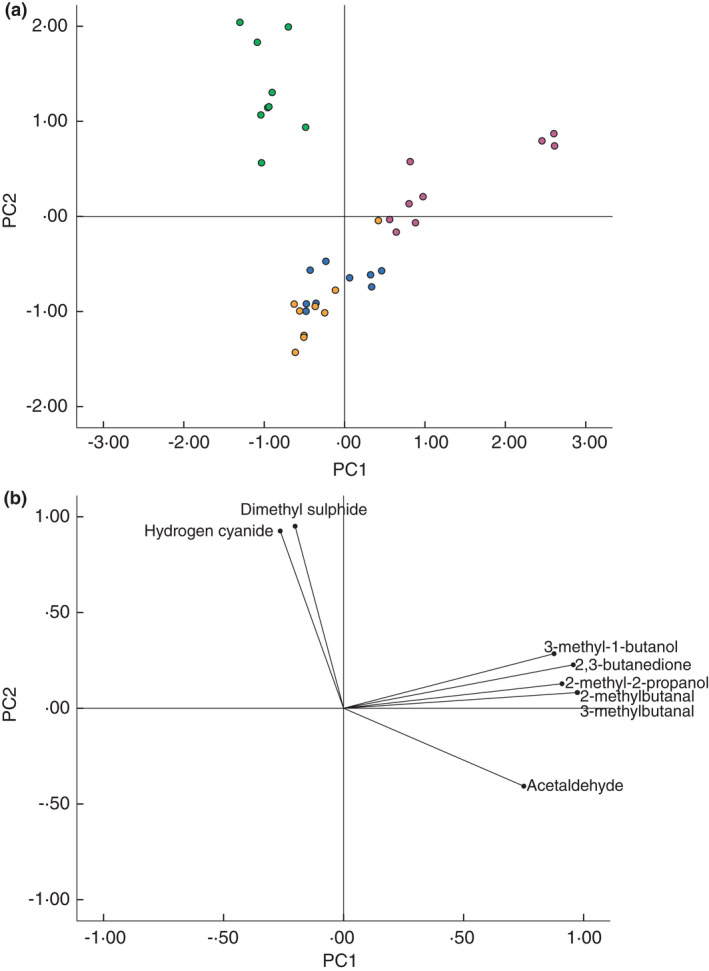
(a) Plot of the scores of the first two principal components generated by principle component analysis of SIFT‐MS SIM scan data of selected headspace volatile compound concentrations (ppb) of bacterial biofilms and uninoculated controls (*n* = 3 scans per sample). Principal component 1 (horizontal axis) accounts for 63.6% of the total variation in the original dataset and principal component 2 (vertical axis) accounts for 26.1% of the total variation. *Pseudomonas aeruginosa* (green), *Staphylococcus aureus* (pink), *Streptococcus pyogenes* (orange), control (blue). (b) Principal component analysis (PCA) loading plot of selected *m*/*z* peaks. Data points indicate the loading of each *m*/*z* peak (variable) on the first two principal components generated from the PCA [Colour figure can be viewed at wileyonlinelibrary.com]

Principal component analysis was used to transform the dataset (eight selected volatile compound concentrations) to principal components, with the inclusion of data from SIFT‐MS SIM analysis of uninoculated controls. Figure 5a shows the scatter plot of the scores of each bacterial biofilm and the uninoculated controls against the first two principal components. Principal components 1 and 2 account for a total of 89.7% of the variability within the dataset of the eight selected volatile compounds. Figure 5a shows that all *P*. *aeruginosa* biofilms occupy a discrete region of the two‐dimensional plot, and one outlier of the *Strep*. *pyogenes biofilm* samples appears within the area of the plot otherwise occupied by *Staph*. *aureus*. As with the analysis of the FS data above, there is also overlap between *Strep*. *pyogenes biofilms* and the ‘background’ volatile compounds detected from uninoculated controls. Figure 5b shows the loading of the eight quantified volatile compounds on the first two principal components and indicates the influence of the production of these compounds on the position of the biofilm samples on the scatter plot shown in Figure 5a. The presence of dimethyl sulphide and hydrogen cyanide seems to most significantly influence the position of the *P*. *aeruginosa* samples on the PCA plot, while the presence of 3‐methyl‐1‐butanol, 2,3‐butanedione, 2‐methyl‐2‐propanol, 2‐methylbutanal, 3‐methylbutanal and acetaldehyde appears to influence the positioning of *Staph*. *aureus* biofilm samples. The absence of these compounds from the headspace of the *Strep*. *pyogenes samples* and controls is the most probable cause of the positioning of these samples on the PCA plot in Figure 5a.

**FIGURE 6:**
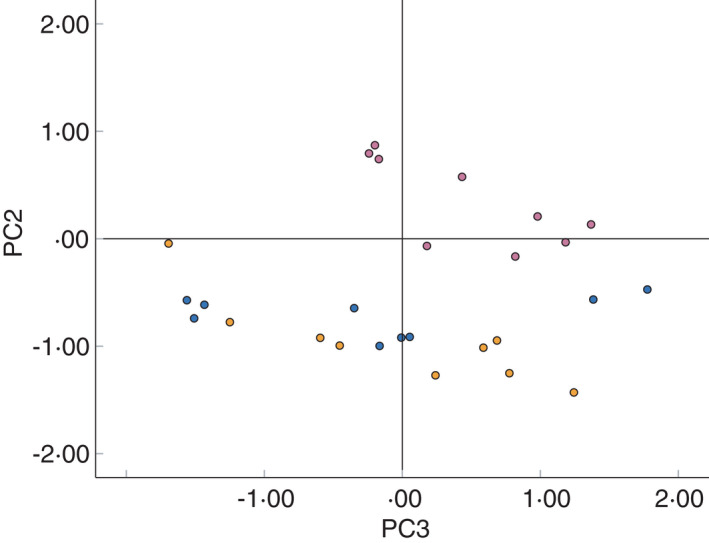
Plot of the scores of the second and third principal components generated by principle component analysis of SIFT‐MS full scan data of selected headspace volatile compound concentrations (ppb). Showing *Staphylococcus aureus* (pink) *Streptococcus pyogenes* (orange) and control (blue) only. Principal component 2 accounts for 26.1% of the variation within the original dataset, while the third components account for 5.45% of the variation [Colour figure can be viewed at wileyonlinelibrary.com]

Figure 6 shows an additional scatter plot which visualizes the scores of the *Strep*. *pyogenes and Staph*. *aureus* biofilms and controls, against the second and third principal components, with principal component 3 accounting for 5.45% of the variation within the dataset. Figure 6 shows that *Strep*. *pyogenes and Staph*. *aureus* separate on the third principal component, but that there is still overlap between *Strep*. *pyogenes and* the controls when the third principal component is employed.

## DISCUSSION

The aim of this work was to determine whether three important bacterial pathogens associated with clinically relevant wound infection, *Staph*. *aureus*, *P*. *aeruginosa* and *Strep*. *pyogenes*, could be successfully discriminated based on their production of volatile metabolites when grown in a collagen wound biofilm model. We have successfully demonstrated that all three species could be differentiated using their characteristic volatile profiles when detected by SIFT‐MS FS and SIM scans (following compound identification using GC‐MS) when combined with the use of multivariate statistical analysis.

For data analysis, MANOVA was applied to select product ions from the mass spectral range (10–200 *m*/*z*) scanned using SIFT‐MS in full scan mode. This resulted in the selection of 59 product ions peaks (*p* < 0.01). Hierarchical cluster analysis of this data shows clear species‐specific clustering of *Staph*. *aureus*, *P*. *aeruginosa* and *Strep*. *pyogenes* biofilms. The dendrogram shown in Figure 1 does not indicate any differences between the different strains of each species analysed. However, MANOVA was used to specifically target product ion peaks likely to drive discrimination between species, and direct observation of the scores within the dissimilarity matrix (not shown) suggests that there are small differences between samples that are not resolved. Incorporating controls into the PCA demonstrates that all three pathogens could be discriminated from background ‘control’ volatiles produced by the collagen model system.

HS‐SPME‐GC‐MS was used to identify compounds in the bacterial biofilm headspace that may be responsible for the product ion peaks detected using SIFT‐MS in full scan mode. In all, 17 compounds were identified from the headspace of the three species included in this work. Other studies (see Table 1) have previously reported the detection of these compounds in the headspace of bacterial cultures (Filipiak et al., 2012; Shestivska et al., 2012; Thorn et al., 2011). The 17 compounds identified by GC‐MS included five alcohols (ethanol, 1‐butanol, isobutyl alcohol, 2‐methyl‐2‐propanol and 3‐methyl‐1‐butanol). Ethanol and 1‐butanol are produced by fermentation of carbohydrates (Thorn & Greenman, 2012) and the branched alcohols are thought to arise from the catabolism of branched amino acids (Audrain et al., 2015). Four aldehydes (acetaldehyde, 2‐methylpropanal, 2‐methylbutanal and 3‐methylbutanal) and four ketones (acetone, acetoin, butanone and 2,3‐butanedione) were also identified by GC‐MS. Catabolism of the amino acid leucine gives rise to 3‐methylbutanal and the alcohol 3‐methyl‐1‐butanol (Filipiak et al., 2012). Catabolism of pyruvate produces acetaldehyde, acetone and acetoin (Filipiak et al., 2012), and oxidation of acetoin results in 2,3‐butanedione production (Audrain et al., 2015). The carboxylic acid, 3‐methylbutanoic acid, identified by GC‐MS, is also produced during catabolism of pyruvate, either from oxidation of 3‐methylbutanal or using an alternative pathway via the intermediate isovaleryl‐CoA (Filipiak et al., 2012). It is suggested that bacterial production of butane may involve cysteine, but the role of this and other amino acids in volatile hydrocarbon synthesis remains unknown (Ladygina et al., 2006). The sulphur compounds detected, dimethyl sulphide and dimethyl disulphide, result from oxidation of methanethiol, which is itself produced from bacterial catabolism of methionine (Audrain et al., 2015; Filipiak et al., 2012).

In addition to the compounds identified by GC‐MS, ammonia and hydrogen cyanide were included for further analysis of bacterial biofilm headspace using SIFT‐MS SIM scans. These compounds have both previously been detected from the headspace of *P*. *aeruginosa*, both in liquid cultures and in vivo (Gilchrist et al., 2013; Neerincx et al., 2015; Smith et al., 2013), with the suggestion that they may be important markers of *P*. *aeruginosa* infection. Hydrogen cyanide is generated through decarboxylation of glycine by the membrane bound HCN synthase enzyme in *P*. *aeruginosa* (Blumer & Haas, 2000), and ammonia is produced by the metabolism of nitrogen containing compounds, including hydrogen cyanide and amino acids (Neerincx et al., 2015).

Previously, studies using SPME have suggested that interaction and competition between compounds in complex mixtures can result in displacement of low molecular weight compounds, by those of higher molecular weights, which can result in under detection of low molecular weight compounds (Murray, 2001). This may explain why ammonia (17 Da) and hydrogen cyanide (28 Da) were not detected during GC‐MS analysis of *P*. *aeruginosa* biofilms, but quantification of these compounds was possible using SIFT‐MS SIM scans. Of the 17 compounds detected by GC‐MS, acetaldehyde has the lowest molecular weight at 44 Da, giving rise to a SIFT‐MS product ion peak of 45 *m*/*z* when using the H_3_O^+^ reagent ion. However, the 59 FS SIFT‐MS product ions selected using MANOVA included seven product ion peaks between 26 and 44 *m*/*z* suggesting additional discriminant compounds of lower molecular weight may be present within the biofilm headspace. One of these product ions, 28 *m*/*z* may result from the production of hydrogen cyanide and is seen at high intensities (cps) in the headspace analysis of all three strains of *P*. *aeruginosa* when observing the raw data (not shown).

Figure 5 shows that of the eight compounds selected using MANOVA, hydrogen cyanide and dimethyl sulphide are driving the position of *P*. *aeruginosa* on the associated PCA plot. Both of these compounds have been identified and quantified in the headspace of *P*. *aeruginosa* culture in a previous study (Shestivska et al., 2012) which used a similar approach, combining GC‐MS and SIFT‐MS for the analysis of *P*. *aeruginosa* cultured using liquid and solid media. In addition, hydrogen cyanide production by *P*. *aeruginosa* has been widely reported in the literature (Askeland & Morrison, 1983; Castric et al., 1979; Gilchrist et al., 2011, 2013; Neerincx et al., 2015; Smith et al., 2013).

The six compounds driving the position of *Staph*. *aureus* on the PCA plot (Figure 5) are as follows: 2‐methyl‐2‐propanol, 3‐methyl‐1‐butanol, acetaldehyde, 2‐methylbutanal, 3‐methylbutanal and 2,3‐butanedione. This suggests high levels of production of these compounds from this particular species. Acetaldehyde and 2‐methylbutanal have been detected in the headspace of *Staph*. *aureus* liquid cultures in previous studies using SIFT‐MS (Chippendale et al., 2011; Thorn et al., 2011), and 2‐methyl‐1‐propanol, 3‐methyl‐1‐butanol, acetaldehyde, 3‐methylbutanal and 2,3‐butanedione have been detected from *Staph*. *aureus* cultures by GC‐MS (Filipiak et al., 2012). Catabolism of pyruvate and leucine gives rise to several of the compounds responsible for the discrimination of *Staph*. *aureus* from the other two species: 3‐methylbutanal and 3‐methyl‐1‐butanol from leucine, and acetaldehyde and 2,3‐butanedione from pyruvate.

Within the *Staph*. *aureus* clusters in both Figures 4 and 5, there appears to be a sub‐group containing the *Staph*. *aureus* NCIMB 6571 biofilms. Scrutiny of the raw data (not shown) indicates that this strain of *Staph*. *aureus* produced several compounds at greater concentrations (by an order of magnitude) than the other strains. For example, 2‐methylbutanal, which has been previously reported in the headspace of *Staph*. *aureus* cultures (Filipiak et al., 2012; Thorn et al., 2011) was measured at >1000 ppb for this strain, compared to between 40 and 110 ppb for the other two strains of *Staph*. *aureus*. Although the compounds produced by the three stains of *Staph*. *aureus* are similar, there appears to be differences in the level of production between strains, which is likely to drive the presence of this sub‐group within the analysis. This suggests strain to strain variation in the activity or production of the metabolic enzymes responsible for generating these metabolites.

There is considerable overlap between *Strep*. *pyogenes* and uninoculated controls in Figure 5, but unlike the FS dataset, plotting the second and third principal components does not aid discrimination of *S*. *pyogenes*. This suggests that use of the specific volatile compounds identified within this work may not enable the discrimination of *Strep*. *pyogenes* from uninoculated controls. However, successful discrimination between *Strep*. *pyogenes* and controls within the FS dataset suggests that this may be possible if additional discriminatory compounds can be identified. Optimization of the GC‐MS methodology, including the use of a variety of SPME fibre materials, may facilitate identification of additional compounds.

A previous study investigated volatile metabolites produced by *Staph*. *aureus* and *P*. *aeruginosa* cultured in TSB using SPME GC‐MS (Filipiak et al., 2012). The authors identified a number of compounds from *Staph*. *aureus* that were detected in the current study, including 3‐methylbutanal, 2‐methylpropanal, 3‐methyl‐1‐butanol, 2,3‐butanedione, 3‐methylbutanoic acid and acetaldehyde (Filipiak et al., 2012). However, they also report many other compounds detected from both *Staph*. *aureus* and *P*. *aeruginosa* that were not detected in the current study. Previously, GC‐MS has been used to investigate the effect of culture media and SPME fibre material on the compounds detected from *Staph*. *aureus* cultures (Tait et al., 2014). Of the compounds detected in the current study, only 3‐methyl‐1‐butanol was detected from cultures of *Staph*. *aureus* in all three culture media used, and 3‐methylbutanal detected using just one type of culture media. Overall, both culture media and SPME fibre material had a significant effect on the compounds detected (Tait et al., 2014).

A recent biofilm study (Ashrafi et al., 2018) used GC‐MS to identify volatile metabolites from *Staph*. *aureus*, *P aeruginosa* and *Strep*. *pyogenes* cultured using a human skin biofilm model. Some of the same compounds found in the current study were identified, including 3‐methylbutanal from *Staph*. *aureus* and hydrogen cyanide from *P*. *aeruginosa*. Identification of ethanol exclusively from *Strep*. *pyogenes* biofilms was also reported, whereas ethanol was detected from all three species within the current study. The investigators also reported a number of compounds that were not detected in the current study. These included 2‐nonanone and 5‐methyl‐2‐hexanone from *P*. *aeruginosa*, 1‐undecene from both *P*. *aeruginosa* and *S*. *pyogenes*, and pentanal from *Staph*. *aureus* (Ashrafi et al., 2018). A plausible reason for these differences is the varying culture conditions and analysis methods employed. Although this previous study and the current study analysed the volatile metabolites released from biofilms of the same three species of wound‐associated bacteria, there are distinct differences between the biofilm models used. The collagen wound biofilm model used in the current study utilizes continuous perfusion of simulated wound fluid to facilitate culture of reproducible steady‐state biofilms (Slade et al., 2019). Whereas in the previous study, human skin was used to provide a representative matrix for biofilm growth within a static model. This would result in accumulation of waste products within the model which were not accounted for. In addition, the use of complex culture media provides a less representative nutrient source (Ashrafi et al., 2018) which, in turn, impacts on the quantity and/or type of metabolites produced.

This study has demonstrated that biofilms of three clinically significant pathogens associated with causing wound infection (*Staph*. *aureus*, *P*. *aeruginosa* and *S*. *pyogenes*) can be differentiated based on real‐time volatile analysis when cultured in a collagen wound biofilm model. Importantly, a comparison of this study with the literature clearly demonstrates that there is a large variation in the range of bacterial volatile compounds detected, dependent on both culture conditions and the analysis method used. However, by using volatile profiling of a select suite of volatiles, differentiation is clearly achievable, as evidenced here. Further studies are required to investigate the production of volatiles under a range of physiological conditions (e.g. varying substrate utilization and growth rate), which is achievable using the collagen wound biofilm model. This would enable a true understanding of the conceivable microbial repertoire of volatile metabolites produced by a given species, including strain to strain variations. In addition, further work should be undertaken on a greater diversity of microorganisms (in mono and mixed culture), including a range of clinical isolates showing antimicrobial resistance or sensitivity. With additional sampling and targeted analysis, it may also be possible to identify differences in the volatile profiles of sensitive and resistant strains to facilitate real‐time identification of antimicrobial susceptibility (Hewett et al. 2020). Ultimately, translation of these findings into clinical practice will require an understanding of both the microbial volatiles produced within the real wound environment and the background volatiles produced by the host (under both healthy and disease conditions). This would help facilitate the future translation of these study findings into clinical practice, either through gas‐sampling of wounds coupled with a centralized analysis facility, or more likely, the development of a suitable low‐cost technology platform to allow detection and interpretation of volatile signatures at the bedside. The potential application of this approach within real‐world scenarios has recently been demonstrated using low‐cost commercially available gas sensors, which were shown to differentially respond to volatiles produced by seven different bacterial species, including *P*. *aeruginosa* (Salinas Alvarez et al. 2019). Therefore, development of this approach in combination with additional volatile compound profiling could produce a diagnostic tool comprised of a gas sensor array targeted to respond to volatile compounds identified specifically for discrimination between species. The resulting diagnostic tool would require in vitro validation against a wide range of clinical microorganisms and strains associated with wound infection, followed by real‐world trials in the clinic.

## CONFLICT OF INTERESTS

None declared.

## Supporting information

Figure S1

## Data Availability

Data is available from the University of the West of England data repository http://researchdata.uwe.ac.uk/613/.
