# Supplementary figures and images for: Real‐time detection of volatile metabolites enabling species‐level discrimination of bacterial biofilms associated with wound infection
Source: J Appl Microbiol. 2021 Oct 19;132(3):1558–72. doi: 10.1111/jam.15313 (PMC9298000; doi:10.1111/jam.15313)

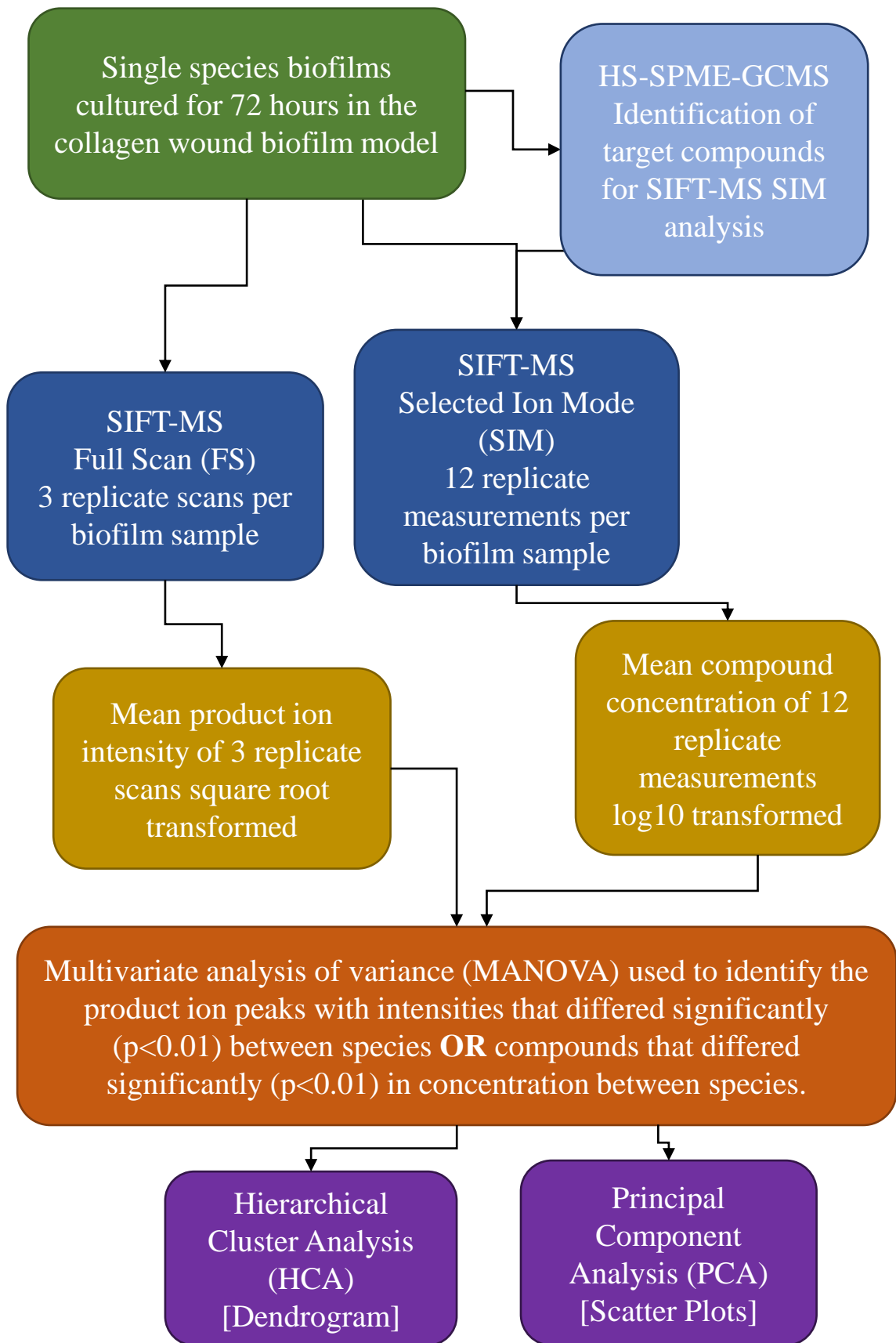

Supplement: Supplementary file 1 — Figure S1 [file JAM-132-1558-s001.pdf]
